# Supplementary material for: Comparison of the intrageneric neutralization scope of monospecific, bispecific/monogeneric and polyspecific/monogeneric antisera raised in horses immunized with sub-Saharan African snake venoms
Source: PLoS Negl Trop Dis. 2024 May 29;18(5):e0012187. doi: 10.1371/journal.pntd.0012187 (PMC11135691; doi:10.1371/journal.pntd.0012187)
Supplement: S1 Table — * Internal reference values for adult creole horses of Instituto Clodomiro Picado. 1 Hematocrit values are expressed as percentage and correspond to the average ± SD (n = 4). 2 Hemoglobin values are expressed as g/dL and correspond to the average ± SD (n = 4). 3 CK: Creatine kinase. Values are expressed as IU/L and correspond to the average ± SD (n = 4). 4 Creatinine. Values are expressed as μmol/L and correspond to the average ± SD (n = 4). 5 Urea. Values are expressed as mg/dL and correspond to the average ± SD (n = 4). 6 AST: Aspartate transaminase. Values are expressed as IU/L and correspond to the average ± SD (n = 4). 7 ALP: Alkaline phosphatase. Values are expressed as IU/L and correspond to the average ± SD (n = 4). 8 GGT: Gamma-glutamyl transferase. Values are expressed as IU/L and correspond to the average ± SD (n = 4). 9 Total protein. Values are expressed as g/dL and correspond to the average ± SD (n = 4). 10 Albumin. Values are expressed as g/dL and correspond to the average ± SD (n = 4). 11 Gamma gap. Values are expressed as g/dL and correspond to the average ± SD (n = 4). (DOC) [file pntd.0012187.s001.doc]

**S1 Table. Hematological and serum chemistry analyses of horses immunized with monogeneric immunogens composed of several venom mixtures. Values correspond to samples collected at the end of each immunization cycle (for monospecific, bispecific and polyspecific antisera).**

| Parameter  (Reference values*) | Immunogen | Antisera specificity | | | | |
| --- | --- | --- | --- | --- | --- | --- |
|  |  | anti  *Bitis* | anti  *Echis* | anti  *Dendroaspis* | anti  Spitting *Naja* | anti  Non spitting *Naja* |
| Hematocrit^1^  (38-50%) | Monospecific | 37.0 ± 6.1 | 40.9 ± 2.3 | 41.2 ± 4.0 | 38.3 ± 4.2 | 37.2 ± 3.8 |
|  | Bispecific | 36.0 ± 5.6 | 39.5 ± 4.8 | 40.0 ± 2.7 | 37.3 ± 3.1 | 35.1 ± 3.2 |
|  | Polyspecific | 34.3 ± 6.7 | 38.9 ± 2.9 | 40.7 ± 5.0 | 35.8 ± 3.7 | 35.3 ± 2.7 |
| Hemoglobin^2^  (13.3-17.9 g/dL) | Monospecific | 12.8 ± 1.7 | 13.9 ± 0.4 | 13.8 ± 1.4 | 13.3 ± 1.6 | 12.8 ± 1.2 |
|  | Bispecific | 12.5 ± 1.9 | 13.6 ± 1.3 | 13.5 ± 1.1 | 12.9 ± 1.0 | 12.3 ± 1.3 |
|  | Polyspecific | 12.6 ± 2.5 | 14.3 ± 1.0 | 14.9 ± 1.8 | 13.1 ± 1.3 | 13.0 ±1.0 |
| CK^3^  (136-401 IU/L) | Monospecific | 343.5 ± 72.9 | 342.2 ± 72.4 | 426.5 ± 210.0 | 641.5 ± 61.9 | 521.5 ± 176.5 |
|  | Bispecific | 320.5 ± 20.5 | 411.3 ± 120.0 | 488.0 ± 250.5 | 399.0 ± 65.9 | 456.0 ± 113.0 |
|  | Polyspecific | 265.0 ± 79.8 | 424.0 ± 121.1 | 431.3 ± 111.4 | 385.0 ± 94.6 | 568.3 ± 383.7 |
| Creatinine^4^  (11-164 μmol/L) | Monospecific | 71.4 ± 12.4 | 75.9 ± 10.5 | 78.2 ± 11.2 | 73.2 ± 1.1 | 78.9 ± 12.7 |
|  | Bispecific | 74.2 ± 13.0 | 81.5 ± 10.1 | 81.5 ± 5.5 | 76.2 ± 11.9 | 82.2 ± 7.7 |
|  | Polyspecific | 74.4 ± 13.2 | 72.4 ± 9.5 | 75.5 ± 12.3 | 73.8 ± 16.0 | 81.8 ± 9.2 |
| Urea^5^  (5.0-12.0 mg/dL) | Monospecific | 5.8 ± 0.4 | 5.0 ± 0.6 | 6.0 ± 1.0 | 5.3 ± 0.4 | 5.8 ± 1.1 |
|  | Bispecific | 6.2 ± 1.1 | 5.9 ± 0.8 | 7.2 ± 1.0 | 6.3 ± 0.4 | 5.9 ± 2.1 |
|  | Polyspecific | 6.8 ± 0.9 | 7.5 ± 1.0 | 7.4 ± 1.2 | 6.8 ± 0.5 | 8.0 ± 0.4 |
| AST^6^  (150-464 IU/L) | Monospecific | 306.7 ± 66.1 | 305.9 ± 59.1 | 312.1 ± 30.4 | 273.5 ± 35.5 | 349.6 ± 69.1 |
|  | Bispecific | 279.6 ± 55.7 | 279.5 ± 47.6 | 331.5 ± 31.1 | 280.0 ± 36.8 | 316.2 ± 31.9 |
|  | Polyspecific | 284.4 ± 39.5 | 312.5 ± 42.1 | 330.9 ± 25.2 | 317.6 ± 60.3 | 375.1 ± 65.9 |
| ALP^7^  (144-433 IU/L) | Monospecific | 405 ± 154 | 252 ± 30 | 353 ± 152 | 351 ± 181 | 350 ± 98 |
|  | Bispecific | 322 ± 108 | 234 ± 25 | 315 ± 83 | 297 ± 107 | 272± 57 |
|  | Polyspecific | 303 ± 96 | 203 ± 32 | 300 ± 106 | 262 ± 90 | 288 ± 84 |
| GGT^8^  (15-49 IU/L) | Monospecific | 17.0 ± 4.8 | 16.0 ± 6.1 | 14.5 ± 5.1 | 16.5 ± 8.9 | 15.5 ± 2.9 |
|  | Bispecific | 17.3 ± 0.5 | 12.7 ± 0.6 | 16.0 ± 5.5 | 13.5 ± 1.3 | 14.8 ± 2.1 |
|  | Polyspecific | 14.8 ± 1.9 | 12.3 ± 1.2 | 14.8 ± 5.1 | 13.0 ± 0.0 | 19.5 ± 11.4 |
| Total protein^9^  (6.3-8.0 g/dL) | Monospecific | 7.4 ± 0.6 | 7.4 ± 0.6 | 7.4 ± 0.4 | 7.6 ± 0.9 | 7.7 ± 0.8 |
|  | Bispecific | 7.7 ± 0.6 | 7.6 ± 0.6 | 7.4 ± 0.2 | 7.6 ± 0.7 | 7.4 ± 0.3 |
|  | Polyspecific | 8.2 ± 1.0 | 7.4 ± 0.6 | 7.6 ± 0.6 | 7.7 ± 1.0 | 7.5 ± 0.4 |
| Albumin^10^  (2.7-3.7 g/dL) | Monospecific | 3.2 ± 0.1 | 3.0 ± 0.1 | 3.0 ± 0.1 | 2.7 ± 0.4 | 3.3 ± 0.7 |
|  | Bispecific | 3.1 ± 0.2 | 3.2 ± 0.1 | 3.1 ± 0.1 | 3.0 ± 0.2 | 3.0 ± 0.1 |
|  | Polyspecific | 3.1 ± 0.2 | 3.2 ± 0.1 | 3.0 ± 0.3 | 2.9 ± 0.2 | 3.0 ± 0.1 |
| Gamma gap^11^  (3.0-4.9 g/dL) | Monospecific | 4.2 ± 0.7 | 4.3 ± 0.7 | 4.5 ± 0.3 | 4.9 ± 0.6 | 4.7 ± 0.7 |
|  | Bispecific | 4.6 ± 0.7 | 4.4 ± 0.5 | 4.3 ± 0.2 | 4.6 ± 0.6 | 4.4 ± 0.3 |
|  | Polyspecific | 5.4 ± 1.0 | 4.3 ± 0.6 | 4.6 ± 0.8 | 4.8 ± 0.8 | 4.5 ± 0.4 |

* Internal reference values for adult creole horses of Instituto Clodomiro Picado.

^1^ Hematocrit values are expressed as percentage and correspond to the average ± SD (n=4).

^2^ Hemoglobin values are expressed as g/dL and correspond to the average ± SD (n=4).

^3^ CK: Creatine kinase. Values are expressed as IU/L and correspond to the average ± SD (n=4).

^4^ Creatinine. Values are expressed as μmol/L and correspond to the average ± SD (n=4).

^5^ Urea. Values are expressed as mg/dL and correspond to the average ± SD (n=4).

^6^ AST: Aspartate transaminase. Values are expressed as IU/L and correspond to the average ± SD (n=4).

^7^ ALP: Alkaline phosphatase. Values are expressed as IU/L and correspond to the average ± SD (n=4).

^8^ GGT: Gamma-glutamyl transferase. Values are expressed as IU/L and correspond to the average ± SD (n=4).

^9^ Total protein. Values are expressed as g/dL and correspond to the average ± SD (n=4).

^10^ Albumin. Values are expressed as g/dL and correspond to the average ± SD (n=4).

^11^ Gamma gap. Values are expressed as g/dL and correspond to the average ± SD (n=4).
