# Supplementary material for: Comparison of the intrageneric neutralization scope of monospecific, bispecific/monogeneric and polyspecific/monogeneric antisera raised in horses immunized with sub-Saharan African snake venoms
Source: PLoS Negl Trop Dis. 2024 May 29;18(5):e0012187. doi: 10.1371/journal.pntd.0012187 (PMC11135691; doi:10.1371/journal.pntd.0012187)
Supplement: S2 Table — *Values correspond to the ED50 and, in parentheses, the corresponding 95% CI. These results were used to construct Figs 2–6. (DOC) [file pntd.0012187.s002.doc]

**S2 Table. Neutralization of the lethality induced in mice by sub-Saharan African venoms, by equine monogeneric antisera*.**

| **Snake venom** | **Monospecific**  **anti-*Bitis***  **antisera** | **Bispecific**  **anti-*Bitis***  **antisera** | **Polyspecific**  **anti-*Bitis***  **antisera** | **Industrial polygeneric plasma** |
| --- | --- | --- | --- | --- |
| *B. gabonica* | 3.4 (2.7-4.4) | 5.0 (3.7-6.9) | 7.8 (6.2-12.0) | 2.0 (1.6-2.9) |
| *B. nasicornis* | 1.2 (0.3-2.5) | 5.2 (3.9-7.4) | 10.3 (7.0-15.6) | 3.4 (2.3-5.8) |
| *B. arietans* | 1.0 (0.5-1.7) | 1.8 (1.1-2.8) | 6.3 (4.0-13.2) | 4.8 (3.6-7.6) |
| *B. rhinoceros* | 6.5 (4.9-8.5) | 8.6 (5.7-14.7) | 12.9 (9.1-18.6) | 2.0 (1.3-3.7) |
|  |  |  |  |  |
| **Snake venom** | **Monospecific**  **anti-*Echis***  **antisera** | **Bispecific**  **anti-*Echis***  **antisera** | **Polyspecific**  **anti-*Echis***  **antisera** | **Industrial polygeneric plasma** |
| *E. leucogaster* | 11.9 (8.3-17.0) | 11.9 (8.3-17.0) | 15.3 (11.9-21.9) | 6.5 (4.7-10.8) |
| *E. ocellatus* | 0.5 (0.-0.8) | 0.9 (0.5-1.4) | 2.3 (1.3-4.3) | 3.3 (1.7-4.4) |
| *E. pyramidum* | 1.4 (1.0-1.8) | 2.3 (1.7-3.5) | 2.6 (2.0-3.4) | 2.4 (1.6-3.9) |
|  |  |  |  |  |
| **Snake venom** | **Monospecific**  **anti-*Dendroaspis***  **antisera** | **Bispecific**  **anti-*Dendroaspis***  **antisera** | **Polyspecific**  **anti-*Dendroaspis***  **antisera** | **Industrial polygeneric plasma** |
| *D. jamesoni* | 0.10 (0.06-0.20) | 0.30 (0.20-0.60) | 0.30 (0.20-0.60) | 0.20 (0.10-0.30) |
| *D. polylepis* | < 0.01 | 0.12 (0.04-0.21) | 0.30 (0.10-0.40) | 0.40 (0.20-0.50) |
| *D. viridis* | 0.05 (0.03-0.11) | 0.08 (0.05-0.11) | 0.40 (0.30-0.60) | 0.40 (0.20-0.50) |
| *D. angusticeps* | 0.20 (0.10-0.40) | 0.40 (0.30-0.70) | 0.50 (0.30-0.80) | 0.20 (0.10-0.40) |
|  |  |  |  |  |
| **Snake venom** | **Monospecific**  **anti-spitting *Naja***  **antisera** | **Bispecific**  **anti-spitting *Naja***  **antisera** | **Polyspecific**  **anti-spitting *Naja***  **antisera** | **Industrial polygeneric plasma** |
| *N. nigricollis* | 2.2 (1.4-3.3) | 3.1 (2.1-4.7) | 4.3 (2.9-7.5) | 0.5 (0.3-0.8) |
| *N. katiensis* | 0.2 (0.1-0.4) | 0.2 (0.1-0.3) | 0.5 (0.1-0.9) | 0.4 (0.2-0.6) |
| *N. ashei* | 1.2 (0.6-1.7) | 1.4 (1.0-2.0) | 1.5 (1.1-2.1) | 0.5 (0.3-0.8) |
| *N. mossambica* | 1.3 (0.6-2.3) | 2.1 (1.2-4.0) | 2.3 (1.5-4.3) | 0.4 (0.2-0.6) |
| *N. nigricincta* | 1.3 (0.6-3.3) | 1.0 (0.5-1.6) | 1.3 (1.0-1.6) | 0.4 (0.2-0.6) |
|  |  |  |  |  |
| **Snake venom** | **Monospecific**  **anti-non-spitting *Naja* antisera** | **Bispecific**  **anti-non- spitting *Naja* antisera** | **Polyspecific**  **anti-non-spitting *Naja* antisera** | **Industrial polygeneric plasma** |
| *N. senegalensis* | 0.3 (0.1-0.5) | 0.7 (0.4-1.0) | 0.6 (0.4-0.9) | 0.2 (0.1-0.3) |
| *N. haje* | 0.4 (0.2-0.7) | 0.6 (0.4-0.9) | 0.6 (0.3-0.8) | 0.1 (0.0-0.2) |
| *N. anchietae* | 0.8 (0.4-1.3) | 1.0 (0.5-2.1) | 1.2 (0.8-1.6) | 0.6 (0.4-0.7) |
| *N. annulifera* | 1.8 (1.0-2.7) | 2.2 (1.6-2.8) | 3.4 (2.3-5.9) | 0.8 (0.6-1.0) |
| *N. melanoleuca* | 0.3 (0.1-0.5) | 0.5 (0.3-0.7) | 1.0 (0.7-1.5) | 0.3 (0.1-0.5) |
| *N. nivea* | 1.0 (0.9-1.1) | 2.2 (1.7-2.8) | 2.3 (2.0-2.8) | 0.5 (0.4-0.8) |

*Values correspond to the ED_50_ and, in parentheses, the corresponding 95% CI. These results were used to construct figures 1-5.
