# Supplementary material for: Comparison of the intrageneric neutralization scope of monospecific, bispecific/monogeneric and polyspecific/monogeneric antisera raised in horses immunized with sub-Saharan African snake venoms
Source: PLoS Negl Trop Dis. 2024 May 29;18(5):e0012187. doi: 10.1371/journal.pntd.0012187 (PMC11135691; doi:10.1371/journal.pntd.0012187)
Supplement: Attachment — Submitted filename: Response to Reviewers.doc [file pntd.0012187.s003.doc]

March 27^th^, 2024

Editors

PLOS Neglected Tropical Diseases.

Dear Editors:

Please find attached the revised version of our manuscript entitled: “Comparison of the intrageneric neutralization scope of monospecific, bispecific/monogeneric and polyspecific/monogeneric antisera raised in horses immunized with sub-Saharan African snake venoms” (Ms. Ref. No.: PNTD-D-24-00197), which had been submitted for publication in PLOS Neglected Tropical Diseases. As we prepared this revised version, we took into consideration all the comments and suggestions made by the reviewers. The modifications and comments on the indications of the reviewers are the following:

--------------------------------------------------------------------------------------------------------------

**Methods:**

*Reviewer #1:*

The methods are detailed and comprehensive.

My main issue with this and the article is that while it is a masterclass for those who are familiar with the field, to be educational to a wider audience including those new to the field and policy makers and future funders for a neglected tropical disease there needs to be education.

To that end, I would suggest the following for consideration:

1. Diagram/Timeline of the inoculation procedure, including the different groups tested...While the methods are dense and detailed, they are hard to make a mental picture.

**Response:** As suggested by the Reviewer, a diagram of the immunization procedure was included in the manuscript (Figure 1; lines 208, 210-215).

2. Pictures of inoculation sites and how they are assessed.

**Response:** We understand the value of the figure suggested by the reviewer. However, since such illustrations have been previously published, we have limited ourselves to stating the corresponding references in the legend of Figure 1 (line 215) and the material and methods section (lines 265-269).

3. There is a reference relating to adjuvants. Can the authors perhaps describe briefly their adjuvant compared to others such as those used in India? What is the advantage of the authors' adjuvant over say glycerine-based adjuvant used elsewhere?

**Response:** Montanide ISA 50 v2 is an immunological adjuvant for veterinary use composed of an injectable mineral oil, vegetable oleic acid and anhydro mannitol ether octodecenoate as emulsifier. After comparing several oil-based adjuvants, we found that Montanide ISA 50 v2 was one of those with the greatest ability to enhance the antibody response of horses towards venoms of African snakes, although it produces some adverse effects at the injection site (Arguedas et al., 2022; doi: 10.1016/j.jvacx.2022.100233). We included some text developing this idea in the materials and methods section (lines 227-231). As we have no experience with glycerin-based adjuvants, we cannot comment on that.

*Reviewer #2:*

Overall, the study objectives are clear and appropriately designed. However, in general the methods are a little too brief (heavily reliant on supplementary for key information that would be included in the main text.

**Response:** As suggested by the Reviewer, we included more details in the description of the methods (lines 179-181, 227-231, 232-236, 249-257, 265-269, 290, 300-301). Moreover, in order to improve the explanation of the immunization of horses, a diagram of the immunization procedure (Figure 1, lines 210-215) was included, and the Table summarizing the immunization scheme was passed from the supplementary material to the main text (lines 239-247).

The methods section could also benefit from the following specific minor revisions;

1. Animal management section - animal acclimatisation period?

**Response:** Before starting the immunization, the horses completed a two-month quarantine during which they were dewormed, acclimatized, and brought to optimal physical condition. This was stated in lines 179-181.

2. More detail regarding the amount of venom given during immunisations. I know a full overview of this is given in supplementary, but this is basic important information I feel should be in the main text.

**Response:** As suggested by the Reviewer, the Table summarizing the immunization scheme was passed from the supplementary material to the main text (lines 239-247).

3. More detail regarding dosing/sampling regime. How long between immunisation dose and sample point etc. Line 207 says "different times" and would benefit from more detail.

**Response:** The lapse between immunization and sampling was 15 days. This was specified in the materials and methods section (lines 232-236).

Lines 2010-2014; a little brief. when were the horses last immunised before plasma harvest for example?

**Response:** According to the suggestion of the Reviewer, more details were included in this part of materials and methods (lines 249-257).

4. ARRIVE guidelines; were mice randomly allocated? were experimenters blinded? gender of cd-1 mice?

**Response:** Mice of both sexes were randomly allocated. Experimenters were not blinded. This was stated in lines 290 and 300-301.

5. Line 246 - typo (2 DL instead of 2 LD)

**Response:** The typo error was corrected (line 293).

*Reviewer #3:*

The methodology used are appropriated and well conducted considering the objective of the manuscript. However, I suggest that the immunization scheme (Table 1) could be showed as the figure 1 to better comprehension of the results.

**Response:** As suggested by the Reviewer, a diagram of the immunization procedure was included in the manuscript (Figure 1; lines 208, 210-215).

--------------------------------------------------------------------------------------------------------------

**Results:**

*Reviewer #1:*

No major concerns

*Reviewer #2:*

Overall, the results and data are clearly presented but could benefit from considering the following specific minor revisions:

1. Lines 269-279 is not really results or discussion, and unnecessary if the methods are detailed enough. I suggest removing and ensuring the immunisation section within the methods is very clear.

**Response:** According to the suggestion of the Reviewer, this paragraph was removed from the manuscript. In order to improve the explanation of the immunization procedure we included: more details in the materials and methods section (lines 227-231 and 232-236), a new figure (Figure 1, lines 210-215), and the Table summarizing the immunization procedure (lines 239-247).

2. Lines 280-283 – which injections of the 120 causes the lesions? All in primary imm with adjuvant? I think the actual imm schedule needs to be in the main manuscript.

**Response:** Just 3.1% of immunogen injections evolved to local tissue lesions. These lesions were associated to the immunogens applied during the first, second and fifteenth weeks, all of which were emulsified on Montanide. This was stated in lines 317-318, 321-322, 329-331. As suggested by the Reviewer, the Table describing the immunization was included in the main manuscript (lines 243-250).

3. Line 314; would be good to state in text the other bitis species that *B. gabonica* neutralised, rather than only having the information in the figure - this was done for the other genera examined.

**Response:** As suggested by the Reviewer, the other *Bitis* species neutralized by the anti-*B. gabonica* serum were stated in the text (line 356).

4. Lines 336-341 – the sentence starts comparing poly to mono, then comments that there were no significant differences between what I presume is poly and bi except for b. arietans – however these stats aren’t described in figure legends (* = vs mono, # = vs industrial). Suggest having a marker for different to bispecific if referring to that comparison in the text (example line 350-352 this exact comparison is remarked on). This should also be applied throughout the manuscript where relevant.

**Response:** The only case in which the difference between bispecific and polyspecific sera was significant is that towards the *B. arietans* venom. An additional marker was included in the figure (now Fig.2) and its legend to refer to this comparison (lines 376-377).

5. Line 354-355 typo/bad gramma, “is the using the”.

**Response:** This error was corrected (lines 402-403).

6. Lines 354-355 – the data isn’t really there to support all 4 – the neutralisation of *B. rhinoceros* was already good with both mono and bi, you could just include the b. ari to improve recognition against that species and stick at 3. But I take the point that it probably doesn’t matter if you include a 4th. It is a minor weakness of the study that it jumps from mono to bi to 4+ for everything except echis as it makes it harder to define the truly important ones in and conclusively say all or just some are needed. But this is more ethical from a 3Rs standpoint.

**Response:** Except for the *E. pyramidum* venom, our experimental design does not allow to determine the contribution to the neutralizing scope made by each venom added to the bispecific immunogens to formulate the polyspecific immunogens. As indicated by the reviewer, testing the addition of each venom involves the use of a large number of horses, which goes against the principles of the 3Rs. In any case, we believe this limitation does not affect the validity of the conclusions of the work.
Additional text developing this idea was introduced in lines 642-648.

7. Line 385-390 - It is interesting that bispecific isn’t sig better than mono for e. oce considering that was what was added in to the immunisation mix. A comment on this with reference to known literature regarding e. oce would improve the discussion element of this section.

**Response**: A comment was added (lines 434-436), suggesting that these findings underscore the antigenic similarity between the venoms of *E. leucogaster* and *E. ocellatus*.

8. Line 436-439 – I dont think the data supports the suggestion that all 4 constitute the best option. I would argue that d. viridis is not needed, this was as effectively neutralised by the industrial blend indicating den pol in the mix is enough, and that the mix of den jam and den pol is enough to neutralise d. angusticeps.

**Response:** To determine how the antibody response induced by African snake venom is affected by other venoms used as co-immunogens, we divide our experimental approach into two phases: 1) In the first phase (i.e., this work), we determine the effect between venoms of the same genus (i.e., intrageneric cross-reactivity/cross-modulation). In this case, the bispecific immunogen composed of venoms of *D. jamesoni* and *D. polylepis* induced an antibody response with low ability to neutralize the venom of *D. viridis*, suggesting that the venom of *D. viridis* has antigens no shared with venoms of *D. jamesoni* and *D. polylepis*. The polygeneric plasma was included just to project the usefulness of the monogeneric experimental antisera in the industrial production of snake antivenoms. 2) In the second phase (i.e., future work), we will determine the effect between venoms of different genera (i.e., intergeneric cross-reactivity/cross-modulation). The inference of the Reviewer corresponds to this second experimental phase, and although reasonable and interesting, it is outside the scope of this work.

9. Line 439-440; Whilst not significantly, there are trends towards this for Den jam and D. ang, and previous discussion for other genra have made use of trends for support of opposing points (line 350-352 for example). Just check use of trends vs significantly different arguments for consistency in supporting data statements.

**Response:** As suggested by the Reviewer, we check the use of trends to support our conclusions. We did not find cases in which trends were used instead of arguments. Only in the case of *Bitis* spp we used a trend to complement the arguments that we use to conclude that the polyspecific immunogen is the best alternative. The arguments were the following: 1) The antibody response induced by the polyspecific immunogen surpassed that of the monospecific immunogen in four venoms while the bispecific just in one, 2) The antibody response induced by the polyspecific immunogen surpasses that of the bispecific immunogen in one venom, 3) There was no venom in which the antibody response induced by the polyspecific immunogen was surpassed by that of the bispecific immunogen, and 4) The antibody response induced by the polyspecific immunogen surpassed that of the industrial immunogen in three venoms while the bispecific just in two.

10. Line 526-527 – there is a large difference between 2 and 6 venoms; I think a comment that the current experimental design makes it hard to infer which no spitting species being added would or wouldn’t increase overall neutralising ability is warranted. Saying bi or poly is quite generic and doesn’t add value to future study designs. Especially as 536-538 suggests adding these would benefit an existing manufactured product - would the authors add just senegalensis and haje, or more?

**Response:** We thank the reviewer for this observation, which is completely valid. The increase in the number of venoms from 2 to 6 in the case of the polyspecific antivenoms anti-non spitting *Naja* venoms does not allow the conclusion of whether fewer venoms will suffice to generate a polyspecific antivenom of wide neutralizing scope in this group of venoms. We have modified the text as to be explicit about this limitation (lines 582-588 and 642-648). The analysis of polyspecific experimental antivenoms using each possible combination of venoms would have demanded a much larger number of horses and mice, and we decided to avoid such possibility. The question remains as to whether other venom combinations, using a lower number of species, would generate a polyspecific antivenom with wide neutralizing scope, an issue that should be tackled in future studies.

11. Figure legends don't refer to mice at all.

**Response:** The fact that the letathality neutralization assay was performed in mice was stated in the figure legends (lines 374, 428, 471, 515 and 562).

12. As a general comment - I think the discussion lacks a bit of depth throughout; it is a very observational discussion. There are some wider discussion points (positive and negative immunomodulation etc), but further venomic data etc could be added throughout to aid discussion and interpretation of results.

**Response:** We have added some sentences in the Discussion related to information previously gathered on venom proteomics and antivenom neutralizing scope of various antivenoms generated with different venom mixtures for immunization (lines 363-367, 390-393, 482-485, 575-576). Also, references [27] and [32] were added.

13. Supp 1 - n numbers missing from legend

The values of n (n = 4) in this supplementary table are indicated in the footnotes of the Table when describing the units used for each determination.

14. Supp 3 table title too brief. Neutralisation of lethality in what? The table alone doesn’t reflect that is it a murine model.

**Response:** The title of this table (now S2 Table) was corrected as suggested by the Reviewer (line 835).

*Reviewer #3:*

Yes, the results are appropriated presented, however some information should be add to the discussion.

**Response:** As indicated in a previous response, we have added several sentences in the Discussion regarding proteomic and immunological information on African venoms which relate to the findings described in this study (lines 363-367, 390-393, 482-485, 575-576). Also, references [27] and [32] were added.

--------------------------------------------------------------------------------------------------------------

**Conclusions:**

*Reviewer #1:*

No major concerns

*Reviewer #2:*

I think overall the observations and subsequent conclusions are well supported with exception to those noted in the results comments above. The limitations are clearly described, although comment could be given to the study design of mono to bi to varying number of species for the poly blend and the difficulties this can cause in drawing conclusions. There is clear evidence for how the work can further understanding of the topic.

**Response:** As indicated in our response to a previous comment, we fully agree with the reviewer in that this is a limitation of our study in terms of the conclusions, especially in the cases where the jump from bispecific to polyspecific antivenom involves more than one additional venom. We have acknowledged this limitation in the new version of our Results and Discussion section (lines 582-588) and Conclusions section (lines 642-648), and we have left the question opened as to whether fewer venoms for the preparation of the polyspecific antivenoms could be enough to generate broad-spectrum antivenoms. The reasons for not doing this, as explained before, is that it would involve a much larger number of horses and mice, which would be quite difficult in terms of the logistics of the study and would be also questionable from the 3Rs perspective.

*Reviewer #3:*

Appropriated.

--------------------------------------------------------------------------------------------------------------

**Summary and general comments:**

*Reviewer #1:*

Very comprehensive work.

*Reviewer #2:*

Authors detail the method for producing antivenom, the medically relevant genera in sSA and the pathology incurred by each, the medically relevant toxins driving pathology, touching on the differences in immunogenicity and host immune response. They refer to a previous rabbit model and detailed species from each genera that had broad intrageneric neutralisation when used as immunogens. Their study uses a mix of venoms from bitis, echis, dendroaspis and naja spp in a scaled up model in horses and look at intrageneric neutralisation of lethality. They clearly demonstrate the changes that including further species within an immunisation mix can cause, and offer opinion on which species of a genre may supplement and enhance existing immunisation methods. The successful translation of work from a previous rabbit study in the same group to a relevant manufacturing model is important. A common thought in the field is that increasing the number of venoms in an immunisation mix to broaden intrageneic neutralisation dilutes the relevant pool of neutralising antibodies against a specific species, and this work provides good evidence that this is not the case.

*Reviewer #3:*

The aim of this manuscript is to contribute with the improvement in the sera production in horses with high ability to neutralize the envenomation by sub-Saharan African snake bites. For this, the authors used an experimental approach for obtaining distinct antivenoms after a scheme of immunization of horses with snake venom combinations according to monospecific, bispecific/monogeneric and polyspecific/monogeneric specificity. The results are consistent showing that the immunization protocols did not induce systemic effect with grade 3 of local lesions in the horses. Furthermore, the data evaluating the capability of distinct antisera to neutralize the lethal activity of the venoms are very interesting. However, to improve the manuscript and understanding the results, some points should be considered and discussed for the publication.

1. The authors should add more information about the industrial polygeneric plasma used in the experiments considering the kinetic of specific-antibody production and the decline of specific-antibody production.

**Response:** A more detailed description of the method followed in the routine immunization of horses for the generation of this hyperimmune plasma has been added to the Materials and Methods section (lines 249-257).

2. The immunization scheme (Table 1) could be showed as the figure 1 to better comprehension of the results.

**Response:** As suggested by the Reviewer, a figure was included in the manuscript in order to improve the description of the immunization procedure (Figure 1; lines 210-215).

3. In general, there is a correlation between the magnitude/affinity the antibody production against antigens/immunogens and the antigenic boosters used to obtain the antisera, how the authors discuss this considering that the time-point of the bleeding to obtain the monospecific, bispecific and polyspecific antisera for comparison the neutralizing potential of each one? The authors have more information or results about this?

**Response:** This is a relevant issue which we have carefully considered and discussed. From our experience on the routine production of antivenoms for Latin America and sub-Saharan Africa, we have observed that, after the initial round of immunization (as the one used to generate the monospecific antisera in this study) the immune response of the horses reaches a plateau, and the level of antibodies, and the neutralizing ability of the hyperimmune plasma, does not improve or increase in further booster immunizations. Usually, the titers reached against a particular venom remains at a similar level during years of re-immunization. On the basis of these previous findings, we can conclude that the improvements in the neutralizing ability of the experimental antisera in our study, when more venoms are added to the immunization mixture, is due to the contribution of ‘new’ venoms to the immunization process and not to an increase in affinity due to repeated venom injections.

4. More information about the diversity and immunogenicity between the venoms of Bitis spp to produce the antisera to reinforce the results and “the increment in the antgenic repertoire when using more venoms”, (lines 343-344, page 14) and also the relevance to add the B.arietans venom to the immunizing mixture to improve the neutralizing ability of the polyspecifc antisera (lines-345-39, page 14), as suggested by the authors in the text.

**Response:** A sentence was added in the section of the Results and Discussion (lines 390-393) corresponding to Bitis sp venoms in which a reference is made to a study that characterized the proteomes of several Bitis sp venoms, highlighting a significant variation in venom composition, which agrees with our observations that a wider neutralizing coverage is obtained when several venoms are used for immunization.

--------------------------------------------------------------------------------------------------------------

**Editorial and data presentation modifications**

*Reviewer #1:*

I would like to see this article focus on educating new readers. This will get published as is, but to be successful it should teach a broader swath of readers. Especially considering the big press on new approaches and some hype about new antibody formats...this communication is critically important. What makes these findings important, interesting and how do they apply to the future of the field and address global shortage?

**Response:** We have added a few sentences in the Abstract, the Introduction and the Conclusions with the aim of making this manuscript more amenable to a general readership, underscoring the relevance of the topic and the findings (lines 52-55, 83-84, 88-89, 111-114, 635-637, 659-663). Also, reference [2] was added.

*Reviewer #2:*

(No Response)

--------------------------------------------------------------------------------------------------------------

We greatly appreciate the contributions of the reviewers and hope this revised version of the manuscript will be suitable for publication in PLOS Neglected Tropical Diseases.

Best regards,

Guillermo León

Instituto Clodomiro Picado

Universidad de Costa Rica
